# CD26-Related Serum Biomarkers: sCD26 Protein, DPP4 Activity, and Anti-CD26 Isotype Levels in a Colorectal Cancer-Screening Context

**DOI:** 10.1155/2020/4347936

**Published:** 2020-01-19

**Authors:** Loretta De Chiara, María Páez de la Cadena, Javier Rodríguez-Berrocal, Mª Carmen Alvarez-Pardiñas, Mª Carmen Pardiñas-Añón, Rubén Varela-Calviño, Oscar J. Cordero

**Affiliations:** ^1^Department of Biochemistry, Genetics and Immunology, Faculty of Biology, University of Vigo, As Lagoas-Marcosende s/n. 36310 Vigo, Spain; ^2^Health Care Unit, Complutense University of Madrid, Spain; ^3^Health Care Unit, University of Santiago de Compostela, Spain; ^4^Department of Biochemistry and Molecular Biology, University of Santiago de Compostela, Santiago de Compostela, Spain

## Abstract

Current screening trials are showing reduction in colorectal cancer incidence and mortality. However, participation rates are often low, and blood-based tests could complement existing screening strategies. CD26 protein (sCD26) and its dipeptidyl peptidase IV (DPP4) enzymatic activity in circulation have been proposed as biomarkers for colorectal cancer and other diseases. However, changes in sCD26 and DPP4 levels show complex degrees of correlation, and their physiological or pathophysiological role is unclear. The aim of this study was to analyse if anti-CD26 autoantibodies are related to sCD26 and DPP4 and to determine their relevance in a context of colorectal cancer screening for complementing the value of sCD26 and DPP4 as biomarkers. These biomarkers were measured in a large prospective cohort (*n* = 497, except the anti-CD26 antibodies, evaluated in 125 samples) that included a subgroup of individuals that were positive for the faecal immunological occult blood test (FIT) (*n* = 86) and underwent a colonoscopy (*n* = 47). We confirmed for the first time higher DPP4 activity in men compared to women (Student's *t* test, *p* = 0.002), though this difference between sexes was not seen for serum sCD26 protein. These biomarkers correlated (*R* = 0.246, *p* = 0.003) only in women. Correlations were found between anti-CD26 isotypes but not with DPP4 activity or sCD26 concentration, except for a negative correlation only in men between anti-CD26 IgA isotype and sCD26 (*R* = −0.232, *p* = 0.044), and an almost significant negative correlation between anti-CD26 IgG and sCD26 limited to FIT-positive men. Interestingly, patients with advanced adenomas displayed the most elevated mean levels of anti-CD26 IgA, IgM, and particularly IgG (Mann-Whitney *U* test, *p* = 0.030) in comparison with the other FIT positives without adenomas, and these levels did not correlate with sCD26 or its DPP4 activity. Our preliminary results suggest that the combination of these measures using sex as confounder could perhaps be used as biomarkers for colorectal disease. It also suggests that events affecting the gut influence the levels of anti-CD26 antibodies, which show little or no effect in antigen clearance. These findings should be confirmed in a larger cohort of individuals with colonoscopy. The physiological origin of the sex differences observed should be further addressed.

## 1. Introduction

Dipeptidyl peptidase IV (DPP4, EC 3.4.14.5, or CD26) is expressed on the surface of both immune and nonimmune (epithelial and endothelial) cell types, as well as a soluble molecule (sCD26) found in biological fluids such as serum [1, 2]. The N-terminal X-Pro cleaving activity from DPP4 regulates chemotactic responses to inflammatory chemokines CCL 3–5, 11, and 22 and CXCL 2 and 9–12, including SDF-1. In addition, it regulates other biologically active peptides such as incretins (GLP-1), neuropeptides, and vasoactive peptides [1–3]. CD26 may also participate in cell signalling [4] and cell infiltration through its nonenzymatic key roles in adhesion and invasion [1, 5, 6].

The role of sCD26 and DPP4 enzymatic activity in biological fluids such as plasma or serum is not clear. Nevertheless, changes in sCD26/DPP4 levels were found in many diseases, suggesting a possible implication in their pathogenesis. Briefly, low levels of DPP4 activity or sCD26 were observed in autoimmunity and immunosuppressed conditions including certain tumours, whereas high levels occur in other tumours, and also in infectious, inflammatory, and liver diseases [1]. We have contributed to the study of sCD26 concentration as a biomarker for early diagnosis and surveillance, mainly in lung cancer and colorectal cancer (CRC) [3, 7–10].

Recently, we have explored DPP4 enzymatic activity and sCD26 in serum from rheumatoid arthritis and uveal melanoma patients and in pleural effusions of benign and malignant lung diseases [10–12], finding complex degrees of correlation including differences between sexes. Several possible arguments could explain the complex relationship between both measures. For example, a low-molecular-weight inhibitor of DPP4 activity was found in sera from patients with metastatic prostate cancer [13], perhaps related to glypican-3, recently reported as a natural inhibitor of CD26/DPP4 enzymatic activity [14], usually absent in adult tissues though expressed in many tumours. Likewise, the variable correlations might be explained by the presence of serum autoantibodies against CD26 [15] we have recently studied [16] and the possibility of Ag-Ab complexes that affect both measures.

As many studies have shown that serum autoantibodies against tumour-associated antigens (TAAs) could serve as potential biomarkers in the detection of several types of cancer [17], the aim of this study was to analyse the relationship between DPP4 activity, sCD26 concentration, anti-CD26 isotypes, and demographic variables, using a prospective cohort of 497 individuals. Among these, the faecal immunological occult blood test (FIT), currently used for CRC screening, was offered to a subgroup of subjects. Based on the colonoscopy result of FIT-positive individuals, we performed a detailed analysis to explore possible improvements in the already known value of sCD26 as a biomarker for CRC.

## 2. Material and Methods

### 2.1. Study Design

This prospective study included a total of 497 subjects: 50 healthy independent donors from the Galician Transfusion Centre (CTG, *Xunta de Galicia*) and 447 individuals that attended the *Servizo de Vixianza da Saúde* (University of Santiago de Compostela, USC, Health Care Unit, HCU) to perform an annual check-up. FIT was offered to individuals aged 40-70 years from the HCU group.

Individuals were divided into the following groups: (a) 50 donors from the CTG, used to check demographic bias in the HCU group; (b) 223 individuals from the HCU with no FIT; (c) 138 individuals from the HCU with a negative FIT; and (d) 86 individuals from the HCU with a positive FIT, used to check the variables under study in the screening context. Sex and age information of the cohort is provided in Table 1.

### 2.2. Ethic Statement

All the procedures described were performed according to clinical ethical practices of the Spanish and European Administrations and approved by the Local Ethics Committee (*Comité Ético de Investigación Clínica de Galicia*, *Xunta de Galicia*, code 2010/298). Written informed consent was obtained from all participants, and anonymity was warranted.

### 2.3. Biological Samples, FIT, and Colonoscopy

For serum collection, peripheral venous blood was extracted with BD SST™ II Advance tubes, allowed to clot at room temperature, and centrifuged at 2,000 *g* for 15 min. Serum was stored at -80°C until use.

The faecal occult blood (ng haemoglobin/mL) was measured using the qualitative immunological test Hem-Check (*Menarini Diagnósticos*, Spain), with a detection range between 0.04 and 120 mgHb/g faeces. Stool samples were collected according to the manufacturer's instructions without specific diet or medication restrictions.

Colonoscopy was offered only to individuals that were positive for FIT. According to colonoscopy results, individuals were classified as no colorectal findings (NCF), benign pathology (BP; including haemorrhoids and diverticula), hyperplastic polyps (HP), adenomas (AD) which include tubular adenomas (TA) and tubulovillous/villous adenomas (TV/V), and CRC.

### 2.4. Measurement of DPP4 Enzyme Activity and Soluble CD26 Protein

DPP4 enzymatic activity was measured in 96-well culture plates using the substrate Gly-Pro-p-nitroanilide (0.2 mM; Sigma-Aldrich, USA), as described previously [10, 12]. The sCD26 concentration was measured in duplicate with the Human DPP4/CD26 DuoSet ELISA kit (R&D Systems, USA) according to the manufacturer's instructions.

### 2.5. Enzyme-Linked Immunosorbent Assays for Anti-CD26 Autoantibodies

We developed an in-house ELISA for the anti-CD26 isotypes IgG, IgM, and IgA [16]. Briefly, concentration of both total and anti-CD26/DPP4 IgA, IgG, and IgM titres in sera of the subjects was determined by ELISA using 96-well cultured plates coated with recombinant sCD26 (rDPP4, 0.5 *μ*g/mL) (R&D Systems, USA) prepared in PBS pH 7.4 and blocked overnight with PBS 0.5% BSA. Plates were incubated with different dilutions of serum for 1 h at 37°C and then washed four times with PBS 0.05% Tween20. Goat anti-human IgM (*μ*-chain), anti IgG (Fab-specific), and IgA (*α*-specific)-peroxidase conjugates (all from Sigma-Aldrich, USA) were used to detect captured antibodies together with standard OPD (o-phenylenediamine dihydrochloride) substrate (Sigma-Aldrich, USA) following the manufacturer's instructions. Absorbance at 450 nm was registered using a Bio-Rad Plate reader (Bio-Rad, USA). Data are shown as absorbance units (Abs). Specificity of the test has been studied before [16].

### 2.6. Statistical Analysis

Descriptive statistics were obtained for continuous (mean and SD) and categorical variables (frequencies). Differences in serum DPP4 activity, sCD26 protein concentration, anti-CD26 IgG, anti-CD26 IgA, and anti-CD26 IgM between two groups were assessed using the parametric Student's *t* test or the nonparametric Mann-Whitney *U* test. The ANOVA test was carried out to compare the variables among more than two groups. Pearson correlation was used to evaluate the strength of the linear relationship between the measured variables. Statistical analyses were carried out with the software SPSS version 20 (SPSS, Chicago IL, USA).

## 3. Results

### 3.1. Levels of Serum DPP4 Activity, sCD26 Concentration, and Anti-CD26 Autoantibody Isotypes according to Demographic Characteristics of the Cohort

Mean and SD levels of the variables measured according to sex and age are shown in Table 2. Men exhibited higher DPP4 activity than women (Student's *t* test, *p* = 0.002), a difference not seen with the concentration of the serum protein (sCD26), in coherence with our previous works [3, 18, 19]. Anti-CD26 Ig isotypes were measured in less samples, finding similar values in both sexes.

In relation to age, no statistically significant differences were found for the measurements among the three age groups (≤50, 51-60, and ≥61 years old). Elder individuals showed a tendency of reduced DPP4 activity and elevated levels of anti-CD26 IgA.

### 3.2. Levels of Serum DPP4 Activity, sCD26 Concentration, and Anti-CD26 Autoantibody Isotypes in the Groups of Donors Defined according to Screening

The tendencies and differences observed in the complete cohort according to sex and age were mainly maintained in both the CTG group and the HCU group (Table 2). Only anti-CD26 IgM levels were significantly lower in the HCU group in relation to the CTG group (Student's *t* test, *p* = 0.005). To elucidate this finding, although the HCU and CTG groups matched age, and men and women showed similar mean values in both groups, IgM levels were analysed separately in the CTG and HCU groups according to sex and age, and we found lower IgM levels in elders (≥61 years) compared to youngers (ANOVA, *p* = 0.084 for HCU and *p* = 0.027 for CTG).

In relation to FIT, no statistically significant differences were found between FIT negatives and FIT positives for any of the measures (Table 2). Likewise, the analyses according to age indicated that both FIT-positive and FIT-negative groups exhibited the same overall tendencies. In contrast to the whole cohort, DPP4 activity was not different between sexes in the FIT-positive and FIT-negative subgroups.

### 3.3. Levels of Serum DPP4 Activity, sCD26 Concentration, and Anti-CD26 Autoantibody Isotypes in the FIT-Positive Group according to the Colonoscopy Findings

Colonoscopy was offered only to individuals with a positive FIT derived from the HCU group, and results of colonoscopy were recorded from 47 subjects. Individuals were classified according to colonoscopy findings as follows: NCF, *n* = 8; BP, *n* = 12; HP, *n* = 6; AD, *n* = 20; and CRC, *n* = 1. All adenomas displayed low-grade dysplasia. The patient with cancer had a tumour in the rectum.

Mean and SD levels of the variables measured according to colonoscopy findings are shown in Table 3. No statistically significant differences were found when we compared each pathological group with NCF and BP, considered as controls or, conservatively, grouping NCF+BP+HP on the one hand and AD+CRC on the other. In both cases, the adenoma group showed the highest levels of DPP4 activity and anti-CD26 isotypes.

To note, the NCF+BP+HP control group showed reduced levels in comparison with the healthy donors of Table 2, in particular IgA and IgM. The Mann-Whitney *U* test performed between these two groups confirmed statistically significant differences in IgA levels (*p* = 0.014) and IgM (*p* = 0.002). The other variables did not show differences between the NCF+BP+HP control group and healthy donors.

Among the 20 adenomas studied, 6 of them had a tubulovillous or villous histology. Interestingly, these lesions, considered in literature as advanced adenomas due to their increased risk of transforming into cancer, displayed the most elevated mean levels of anti-CD26 IgA (0.32 ± 0.12 Abs), IgG (0.33 ± 0.06 Abs), and IgM (0.23 ± 0.13 Abs) (NCF+BP+HP *vs.* AA, Mann-Whitney *U* test *p* = 0.098, *p* = 0.030, and *p* = 0.143, respectively).

**Figure 1:**
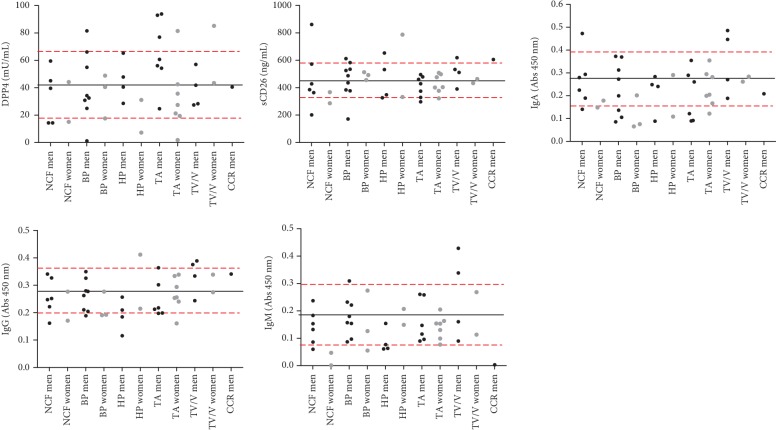
Levels of DPP4 activity, sCD26 protein, and anti-CD26 IgA, IgG, and IgM according to the colonoscopy findings in men and women. Legend: NCF: no colorectal findings; BP: benign pathology; HP: hyperplasic polyp; TA: tubular adenomas; TV/V: tubulovillous/villous adenomas; CRC: colorectal cancer. Black line represents the mean measurement in the entire cohort; red dashed lines represent the standard deviation.

Statistical analyses were not performed separating men from women due to the small number of individuals in each group. However, we represented all data according to the colonoscopy findings in men and women (Figure 1), and the highest levels of anti-CD26 isotypes were found in some men with tubulovillous or villous histology.

### 3.4. Correlations of Anti-CD26/DPP4 Autoantibody Isotype Levels, Serum DPP4 Activity, and sCD26 Concentrations

**Figure 2:**
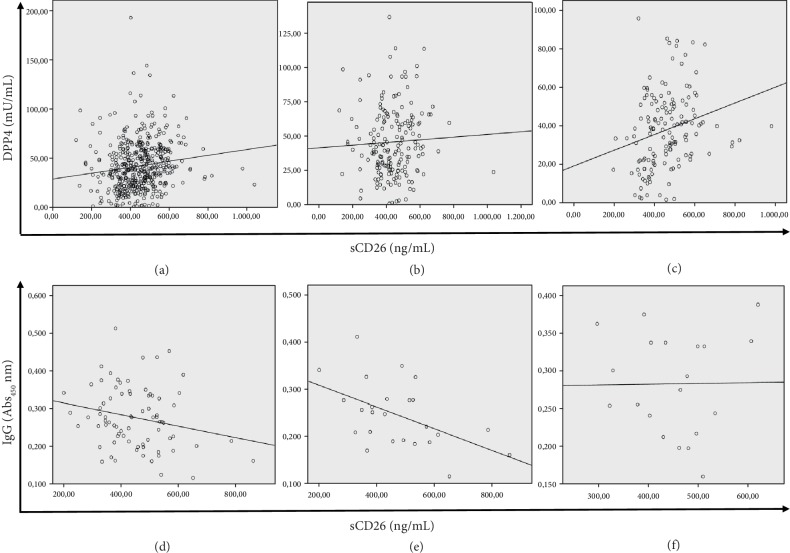
Correlation scatterplots with linear regression line. In the upper row, sCD26 vs. DPP4 in (a) the entire cohort, (b) men, and (c) women. In the lower row, sCD26 vs. IgG in (d) FIT positives, (e) individuals with no neoplasia (no colorectal finding+benign pathology+hyperplastic polyps), and (f) individuals with neoplasia (adenomas+cancer).

Correlations among these variables were first assessed in the whole cohort (Table 4) and then separately in women (Supplementary [Supplementary-material supplementary-material-1]), in men (Supplementary [Supplementary-material supplementary-material-1]), and in the three age groups (Supplementary Tables [Supplementary-material supplementary-material-1]), due to the previous results. In the whole cohort, the correlation found between DPP4 and sCD26 was positive though weak (*R* = 0.138, *p* = 0.003, Figure 2(a) upper row). Anti-CD26 isotypes significantly correlated among them, but only IgA showed a trend to negative correlation with sCD26 levels (*R* = −0.149, *p* = 0.084).

In men, while the correlations among isotypes remained, sCD26 and DPP4 did not correlate (Supplementary [Supplementary-material supplementary-material-1], Figure 2(b) upper row). However, the negative correlation of anti-CD26 IgA *vs.* sCD26 was evident (*R* = −0.232, *p* = 0.044). In women, the correlation between DPP4 and sCD26 was slightly higher (*R* = 0.246, *p* = 0.003, Figure 2(c) upper row), and most correlations among isotypes persisted (Supplementary [Supplementary-material supplementary-material-1]).

When correlations were analysed individually in each age group, the positive correlation between DPP4 and sCD26 persisted in the younger aged groups (≤50 and 51-60 years) but was absent among the elder individuals (≥61 years) (Supplementary Tables [Supplementary-material supplementary-material-1]). These analyses also revealed a new correlation between DPP4 activity *vs.* anti-CD26 IgG that largely changed according to age; no correlation was found in the age group ≤ 50 years, a negative correlation appeared in the 51-60 years group (*R* = −0.347, *p* = 0.028), and then a positive correlation was evidenced in ≥61 years individuals (*R* = 0.386, *p* = 0.038).

In addition, correlation analyses were also performed separately in FIT-positive and FIT-negative groups and in the 47 individuals with a colorectal diagnosis. The DPP4 *vs.* sCD26 correlation was lost in FIT positives and the subgroups with pathological colonoscopy findings (data not shown). FIT positives showed an almost significant negative correlation between anti-CD26 IgG and sCD26 (*R* = −0.220, *p* = 0.054, Figure 2(d) lower row), a trend limited to the ≤50 age group. Interestingly, in the nonadenoma group that included NCF, BP, and HP, IgG correlated negatively with its antigen sCD26 (*R* = −0.348, *p* = 0.019, Figure 2(e) lower row) and almost correlated with DPP4 activity (*R* = −0.284, *p* = 0.062). However, the adenoma and CRC groups, while maintaining the positive correlations between the three isotypes, lacked correlation between any anti-CD26 and sCD26 or its DPP4 activity (Figure 2(f) lower row).

### 3.5. Study of the Diagnostic Possibilities of DPP4 Activity, sCD26 Concentration, and Anti-CD26 Autoantibody Isotypes in Men and Women

We have reported that the combination of serum sCD26 and the faecal blood test could result a valuable strategy for detecting advanced neoplasia in CRC screening [8].

The facts that the advanced adenomas showed the highest levels of the anti-CD26 isotypes and that their values do not correlate in this group with the other biomarkers might support the combined use of all these biomarkers for early diagnosis of malignancy if they enhanced the number of positively diagnosed for that condition.

Figure 1 shows the values of DPP4, sCD26, and anti-CD26 IgA, IgG, and IgM according to the colonoscopy findings and also contains cut-offs to define biomarker positivity. In this prospective study, the cut-offs were set according to the mean ± SD of the whole cohort, based solely on the population distribution due to the small sample size. The outliers or cases with levels above or below the mean + SD or −SD, respectively, were considered positives. These cut-offs for each biomarker are represented with dashed red lines in Figure 1. According to this simple analysis, a larger number of adenoma and CRC cases resulted below or above the proposed cut-offs for anti-CD26 isotypes compared to sCD26. Therefore, it seems possible that the anti-CD26 isotypes may perform better than sCD26 for detecting advanced neoplasia.

## 4. Discussion

The first important result in this study was that men showed statistically higher serum DPP4 activity than women, confirming the trend suggested in an early study [18]. This difference between sexes was not seen with the concentration of the serum protein (sCD26), in coherence with what others and we have reported several times [10–12, 19]. On the other hand, DPP4 activity and sCD26 correlated in females but not in males, so there is obviously an unknown factor affecting this protein related to sex.

Serum and plasma DPP4 activity in healthy men was slightly higher than that in healthy women at any point of the menstrual cycle in a study that did not find significant association between plasma DPP4 enzymatic activity and sex [18]. However, in that study, DPP4 activity decreased significantly with age. Therefore, the fact that serum DPP4 activity was found statistically lower in men than women in a cohort similarly large as ours can be explained because it is older [20].

As plasma sCD26 concentration, measured with the same kit we used, was higher in men than in women among healthy controls [21] (although it was not in multiple sclerosis (MS) patients), the difference between plasma and serum protein concentration in relation to sex suggests that a component of the coagulation process in serum might be interfering with the epitope used in the ELISA. It should be tested whether plasminogen [15, 22] or fibrinogen [10], which have been found bound to the CD26 protein, is responsible for this variation. However, the finding that DPP4 activity correlated with sCD26 in women but not in men might suggest, alternatively, that this putative product of the coagulation system or another activator might be enhancing serum DPP4 enzymatic activity in the latter. In fact, the values of serum DPP4 activity are higher than those in plasma [18].

The physiological meaning of this fact is currently unknown, although its importance is supported because this weak correlation found in women (more in youngers) has been found enhanced in patients under certain immunotherapies in autoimmune disease [11]. In addition, the use of DPP4 inhibitor sitagliptin, alone or together with enalaprilat during ACE (angiotensin-converting-enzyme) inhibition [23], diminished substance P-dependent tissue plasminogen activator release only in women.

The correlation sCD26 *vs.* DPP4 found in women does not support, however, an effect of andropause/menopause on gene expression, which has been observed for other peptidases similar to DPP4 [24]. Intriguingly, although DPP4 is not related to the renin-angiotensin system like those peptidases, it affects also blood pressure through some of its physiological substrates [1, 18, 25].

However, a factor related to hormonal regulation, not directly affecting DPP4 gene expression, cannot be excluded. For example, it was observed that circulating levels of 25(OH)-vitamin D showed a negative correlation with DPP4 activity in MS patients, particularly in women, suggesting that vitamin D may affect DPP4 activity differently in women and men with MS [21].

Here, we checked the relationship of anti-CD26 Abs with the other biomarkers, taking into account that antiplasminogen levels, in particular the IgG isotype, were recently related to some tumours [26]. The only hypothesis about the origin of anti-CD26 autoantibodies in healthy controls links again CD26/DPP4 with plasminogen as a consequence of an abnormal immune stimulation triggered by streptokinase (SK) released during streptococcal infections, a mechanism that also produces antiplasminogen [15, 22]. These authors as well as us [16] have found elevated levels of anti-CD26 Abs in some autoimmune diseases.

Apparently, as none of the anti-CD26 values correlated with serum DPP4 activity or sCD26 concentrations in the cohort of healthy donors, their function is not related to antigen clearance, as was first suggested by Cuchacovich et al. [15] but was not confirmed in another cohort [16]. However, the significant or near significant data for negative correlations between IgA and IgG and their Ag found in men suggests that a basal effect on sCD26 levels cannot be discarded. Intriguingly, the same trend was not seen in women.

Because of these or other facts, a few more individuals with colonoscopy showed more anomalous values for DPP4 than for sCD26. However, our most remarkable finding was the large number of individuals with anomalous anti-CD26 levels, in particular with lower IgA values in both men and women with different benign colorectal pathologies than the whole or the healthy cohorts. On the contrary, the group with tubulovillous/villous adenomas exhibited higher titres of anti-CD26 (statistically significant for the IgG isotype, the one independent of sex and age) together with high levels of DPP4 and low levels of sCD26.

This study has several limitations. First, the measurement of the anti-CD26 isotypes was not possible in all the samples included in the study, losing statistical power in some group comparisons. Second, information regarding age and sex was not registered in some of the individuals. Third, the analyses regarding the subcohort of FIT-positive individuals with colonoscopy include a small number of individuals when grouped according to pathology, especially for individuals with tubulovillous/villous adenomas and cancer. This prevents us from performing more statistical analyses and drawing more solid conclusions.

If it can be demonstrated that events in the gut are affecting the normal levels of these anti-CD26 Abs, the eventual pathophysiological role associated with them must be investigated. Some TAAs are absent or expressed at very low levels in healthy donors, and higher antibody titres are reported when tumours expressed the respective TAA [27]. This is not the case of CD26 in CRC, at least in earlier stages where CRC tumours lose the abundant epithelial CD26 expression. Importantly, autoantibodies against autologous TAA are detected in the asymptomatic stage of cancer and can thus serve as biomarkers for early cancer diagnosis [28]. If the autoantibodies were already present, the changes would still be detected earlier since it is not necessary to trigger the whole humoural mechanism. It may be relevant that high antiplasminogen levels have been found in the tumours analysed (though no information was given for colorectal tumours [26]). Anti-CD26 might then participate in autoantibody signatures that would aid in the development of diagnostics, prognostics, and follow-up of therapeutics for cancer patients [27, 28] and perhaps could identify subgroups of patients for precision medicine. Autoantibodies are also a potential therapeutic target for cancer therapy, and some of them are already being used in immunotherapy [29, 30].

## 5. Conclusion

This prospective work supports further studies to unravel the differences between antigen sCD26 and DPP4 activity according to sex, to know how the anti-CD26 Abs are generated, if they play a pathological role and if they may help clinical daily practice (like their antigen sCD26 [8, 9]).

## Figures and Tables

**Table 1:** Characteristics of the individuals included in the cohort.

	CTG group (*n* = 50)	HCU group		
		No FIT^1^ (*n* = 223)	Negative for FIT (*n* = 138)	Positive for FIT (*n* = 86)
Sex				
Men	21 (42.0%)	42 (18.8%)	74 (53.6%)	53 (61.6%)
Women	29 (58.0%)	20 (9.0%)	64 (46.4%)	33 (38.4%)
Age				
≤50 years	27 (54.0%)	—	67 (48.6%)	34 (39.5%)
51-60 years	16 (32.0%)	—	50 (36.2%)	29 (33.7%)
≥61 years	7 (14.0%)	—	21 (15.2%)	23 (26.7%)

FIT: faecal immunochemical test. ^1^Sex information is missing from 161 individuals, while age is missing for all the individuals from the no FIT group.

**Table 2:** Levels of serum DPP-IV activity, sCD26 concentration, and anti-CD26/DPP-IV autoantibody isotypes according to demographic characteristics and origin of the cohort.

Characteristic	DPP4 Mean ± SD (mU/mL)			sCD26 Mean ± SD (ng/mL)			IgA Mean ± SD (Abs_450nm_)			IgG Mean ± SD (Abs_450nm_)			IgM Mean ± SD (Abs_450nm_)		
	*N*		*p* value	*N*		*p* value	*N*		*p* value	*N*		*p* value	*N*		*p* value
Sex															
Men	182	45.20 ± 24.87	**0.002** ^a^	189	443.19 ± 129.39	0.176^a^	76	0.26 ± 0.12	0.530^a^	76	0.27 ± 0.08	0.328^a^	76	0.17 ± 0.09	0.146^a^
Women	146	37.64 ± 19.69		144	461.87 ± 117.87		48	0.28 ± 0.11		49	0.29 ± 0.08		49	0.20 ± 0.13	
Age (years)															
≤50	126	39.98 ± 22.61		126	452.22 ± 110.88		54	0.25 ± 0.10		55	0.27 ± 0.08		55	0.18 ± 0.08	
51-60	95	41.55 ± 18.85	0.206^b^	94	458.03 ± 98.92	0.494^b^	40	0.26 ± 0.11	0.062^b^	40	0.29 ± 0.10	0.716^b^	40	0.19 ± 0.14	0.296^b^
≥61	50	35.15 ± 19.35		51	436.68 ± 94.06		29	0.31 ± 0.13		29	0.27 ± 0.07		29	0.15 ± 0.10	
Group															
CTG (*n* = 50)	50	43.51 ± 15.86	0.542^a^	50	459.50 ± 103.48	0.528^a^	25	0.28 ± 0.08	0.863^a^	25	0.27 ± 0.10	0.673^a^	25	0.24 ± 0.15	**0.005** ^a^
HCU (*n* = 447)	434	41.95 ± 25.02		443	448.58 ± 117.37		112	0.28 ± 0.13		113	0.28 ± 0.08		113	0.17 ± 0.09	
HCU group relative to FIT															
Negative for FIT (*n* = 138)	137	37.44 ± 22.13	0.249^a^	135	451.98 ± 95.21	0.652^a^	22	0.28 ± 0.11	0.535^a^	23	0.28 ± 0.08	0.851^a^	23	0.17 ± 0.08	0.842^a^
Positive for FIT (*n* = 86)	84	40.92 ± 20.97		86	445.49 ± 116.75		77	0.26 ± 0.12		77	0.28 ± 0.08		77	0.16 ± 0.09	

FIT: faecal immunochemical test; ^a^Student's *t* test; ^b^ANOVA test.

**Table 3:** Levels of serum DPP-IV activity, sCD26 concentration, and anti-CD26/DPP-IV autoantibody isotypes according to the colonoscopy result in FIT-positive individuals.

Colonoscopy finding	DPP4 Mean ± SD (mU/mL)			sCD26 Mean ± SD (ng/mL)			IgA Mean ± SD (Abs_450nm_)			IgG Mean ± SD (Abs_450nm_)			IgM Mean ± SD (Abs_450nm_)		
	*N*		*p* value^1^	*N*		*p* value^1^	*N*		*p* value^1^	*N*		*p* value^1^	*N*		*p* value^1^
NCF	7	33.19 ± 18.40		8	433.96 ± 203.46		8	0.24 ± 0.11		8	0.25 ± 0.07		8	0.11 ± 0.08	
BP	12	39.90 ± 21.62		12	465.20 ± 116.50		11	0.20 ± 0.12		11	0.25 ± 0.06		11	0.17 ± 0.08	
HP	6	36.78 ± 19.57	0.924	6	497.18 ± 193.87	0.903	6	0.21 ± 0.09	0.899	6	0.23 ± 0.10	0.408	6	0.12 ± 0.06	0.484
AD	20	48.64 ± 26.63	0.227	20	440.76 ± 79.77	0.808	19	0.25 ± 0.11	0.365	19	0.28 ± 0.07	0.184	19	0.18 ± 0.09	0.439
CRC	1	40.52		1	605.93		1	0.21		1	0.34		1	0.003	
NCF+BP+HP	25	37.27 ± 19.67		26	462.97 ± 160.10		25	0.22 ± 0.11		25	0.25 ± 0.07		25	0.14 ± 0.08	
AD+CRC	21	48.25 ± 26.01	0.221^2^	21	448.62 ± 85.70	0.898^2^	20	0.25 ± 0.11	0.309^2^	20	0.28 ± 0.07	0.083^2^	20	0.17 ± 0.1	0.385^2^

NCF: no colorectal finding; BP: benign pathology; HP: hyperplastic polyp; AD: adenomas; CRC: colorectal cancer; ^1^Mann-Whitney *U* test for comparison of NCF+BP *vs.* the other groups; ^2^Mann-Whitney *U* test for comparison of NCF+BP+HP *vs.* AD+CRC.

**Table 4:** Correlation of DPP-IV, sCD26, IgA, IgG, and IgM in all the cohort.

	DPP4	sCD26	IgA	IgG	IgM
DPP4 *R*	1	**0.138** ^∗∗^	0.038	-0.015	0.003
*p* value		**0.003**	0.661	0.867	0.968
*N*	484	480	134	135	135
	sCD26 *R*	1	-0.149	-0.117	-0.021
	*p* value		0.084	0.174	0.803
	*N*	493	136	137	137
		IgA *R*	1	**0.441** ^∗∗^	**0.268** ^∗∗^
		*p* value		**<0.0001**	**0.002**
		*N*	136	136	136
			IgG *R*	1	**0.638** ^∗∗^
			*p* value		**<0.0001**
			*N*	137	137
				IgM *R*	1
				*p* value	
				*N*	137

*R*: Pearson correlation coefficient.

## Data Availability

The datasets analysed during the current study are available from the corresponding author on reasonable request.

## Supplementary Materials

Supplementary Materials Supplementary Table 1: correlation of DPP-IV, sCD26, IgA, IgG, and IgM in women from the complete cohort. Supplementary Table 2: correlation of DPP-IV, sCD26, IgA, IgG, and IgM in men from the complete cohort. Supplementary Table 3: correlation of DPP-IV, sCD26, IgA, IgG, and IgM in age group ≤ 50 years from the complete cohort. Supplementary Table 4: correlation of DPP-IV, sCD26, IgA, IgG, and IgM in age group 51-60 years from the complete cohort. Supplementary Table 5: correlation of DPP-IV, sCD26, IgA, IgG, and IgM in age group ≥ 61 years from the complete cohort.

## References

[1] Cordero O. J., Salgado F. J., Nogueira M. (2009). On the origin of serum CD26 and its altered concentration in cancer patients. *Cancer Immunology, Immunotherapy*.

[2] Proost P., Mahieu F., Schutyser E., Van Damme J. (2004). Posttranslational processing of chemokines. *Methods in Molecular Biology*.

[3] Cordero O. J., Imbernon M., Chiara L. D. (2011). Potential of soluble CD26 as a serum marker for colorectal cancer detection. *World Journal of Clinical Oncology*.

[4] Muscat C., Bertotto A., Agea E. (1994). Expression and functional role of 1F7 (CD26) antigen on peripheral blood and synovial fluid T cells in rheumatoid arthritis patients. *Clinical and Experimental Immunology*.

[5] Havre P. A., Dang L. H., Ohnuma K., Iwata S., Morimoto C., Dang N. H. (2013). CD26 expression on T-anaplastic large cell lymphoma (ALCL) line Karpas 299 is associated with increased expression of versican and MT1-MMP and enhanced adhesion. *BMC Cancer*.

[6] Havre P. A., Abe M., Urasaki Y., Ohnuma K., Morimoto C., Dang N. H. (2009). CD26 expression on T cell lines increases SDF-1-*α*-mediated invasion. *British Journal of Cancer*.

[7] Blanco-Prieto S., Vázquez-Iglesias L., Rodríguez-Girondo M. (2015). Serum calprotectin, CD26 and EGF to establish a panel for the diagnosis of lung cancer. *PLoS One*.

[8] Otero-Estévez O., de Chiara L., Rodríguez-Berrocal F. J. (2015). Serum sCD26 for colorectal cancer screening in family-risk individuals: comparison with faecal immunochemical test. *British Journal of Cancer*.

[9] De Chiara L., Rodríguez-Piñeiro A. M., Cordero O. J. (2014). Postoperative serum levels of sCD26 for surveillance in colorectal cancer patients. *PLoS One*.

[10] Sánchez-Otero N., Rodríguez-Berrocal F. J., de la Cadena M. P., Botana-Rial M. I., Cordero O. J. (2015). Evaluation of pleural effusion sCD26 and DPP-IV as diagnostic biomarkers in lung disease. *Scientific Reports*.

[11] Cordero O. J., Varela-Calviño R., López-González T. (2015). CD26 expression on T helper populations and sCD26 serum levels in patients with rheumatoid arthritis. *PLoS One*.

[12] Varela-Calviño R., Imbernón M., Vázquez-Iglesias L. (2015). Serum dipeptidyl peptidase IV activity and sCD26 concentration in patients with choroidal nevus or uveal melanoma. *Clinica Chimica Acta*.

[13] Nazarian A., Lawlor K., Yi S. S. (2014). Inhibition of circulating dipeptidyl peptidase 4 activity in patients with metastatic prostate cancer. *Molecular & Cellular Proteomics*.

[14] Davoodi J., Kelly J., Gendron N. H., MacKenzie A. E. (2007). The Simpson-Golabi-Behmel syndrome causative glypican-3, binds to and inhibits the dipeptidyl peptidase activity of CD26. *Proteomics*.

[15] Cuchacovich M., Gatica H., Pizzo S. V., Gonzalez-Gronow M. (2001). Characterization of human serum dipeptidyl peptidase IV (CD26) and analysis of its autoantibodies in patients with rheumatoid arthritis and other autoimmune diseases. *Clinical and Experimental Rheumatology*.

[16] Cordero O. J., Varela-Calviño R., López-González T. (2017). Anti-CD26 autoantibodies are involved in rheumatoid arthritis and show potential clinical interest. *Clinical Biochemistry*.

[17] Zhang H. F., Qin J. J., Ren P. F. (2016). A panel of autoantibodies against multiple tumor-associated antigens in the immunodiagnosis of esophageal squamous cell cancer. *Cancer Immunology, Immunotherapy*.

[18] De Chiara L., Rodríguez-Piñeiro A. M., Cordero O. J. (2009). Soluble CD26 levels and its association to epidemiologic parameters in a sample population. *Disease Markers*.

[19] Durinx C., Neels H., Van der Auwera J. C., Naelaerts K., Scharpe S., De Meester I. (2001). Reference values for plasma dipeptidyl-peptidase IV activity and their association with other laboratory parameters. *Clinical Chemistry and Laboratory Medicine*.

[20] Sanz B., Larrinaga G., Fernandez-Atucha A. (2018). Obesity parameters, physical activity, and physical fitness are correlated with serum dipeptidyl peptidase IV activity in a healthy population. *Heliyon*.

[21] Tejera-Alhambra M., Casrouge A., de Andrés C. (2014). Low DPP4 expression and activity in multiple sclerosis. *Clinical Immunology*.

[22] Cuchacovich M., Gatica H., Vial P., Yovanovich J., Pizzo S. V., González-Gronow M. (2002). Streptokinase promotes development of dipeptidyl peptidase IV (CD26) autoantibodies after fibrinolytic therapy in myocardial infarction patients. *Clinical and Diagnostic Laboratory Immunology*.

[23] Devin J. K., Pretorius M., Nian H., Yu C., Billings F. T., Brown N. J. (2014). Substance P increases sympathetic activity during combined angiotensin-converting enzyme and dipeptidyl peptidase-4 inhibition. *Hypertension*.

[24] Fernández-Atucha A., Izagirre A., Fraile-Bermúdez A. B. (2017). Sex differences in the aging pattern of renin-angiotensin system serum peptidases. *Biology of Sex Differences*.

[25] Jackson E. K., Dubinion J. H., Mi Z. (2008). Effects of dipeptidyl peptidase IV inhibition on arterial blood pressure. *Clinical and Experimental Pharmacology & Physiology*.

[26] Goufman E. I., Yakovlev V. N., Tikhonova N. B. (2015). Autoantibodies to plasminogen and their role in tumor diseases. *Bulletin of Experimental Biology and Medicine*.

[27] Reuschenbach M., von Knebel Doeberitz M., Wentzensen N. (2009). A systematic review of humoral immune responses against tumor antigens. *Cancer Immunology, Immunotherapy*.

[28] Tan H. T., Low J., Lim S. G., Chung M. C. (2009). Serum autoantibodies as biomarkers for early cancer detection. *The FEBS Journal*.

[29] Brändlein S., Pohle T., Ruoff N., Wozniak E., Müller-Hermelink H. K., Vollmers H. P. (2003). Natural IgM antibodies and immunosurveillance mechanisms against epithelial cancer cells in humans. *Cancer Research*.

[30] Cohen I. R. (2014). Activation of benign autoimmunity as both tumor and autoimmune disease immunotherapy: a comprehensive review. *Journal of Autoimmunity*.

