# Supplementary material for: CD26-Related Serum Biomarkers: sCD26 Protein, DPP4 Activity, and Anti-CD26 Isotype Levels in a Colorectal Cancer-Screening Context
Source: Dis Markers. 2020 Jan 19;2020:4347936. doi: 10.1155/2020/4347936 (PMC6995486; doi:10.1155/2020/4347936)
Supplement: Supplementary Materials — Supplementary Table 1: correlation of DPP-IV, sCD26, IgA, IgG, and IgM in women from the complete cohort. Supplementary Table 2: correlation of DPP-IV, sCD26, IgA, IgG, and IgM in men from the complete cohort. Supplementary Table 3: correlation of DPP-IV, sCD26, IgA, IgG, and IgM in age group ≤ 50 years from the complete cohort. Supplementary Table 4: correlation of DPP-IV, sCD26, IgA, IgG, and IgM in age group 51-60 years from the complete cohort. Supplementary Table 5: correlation of DPP-IV, sCD26, IgA, IgG, and IgM in age group ≥ 61 years from the complete cohort. [file 4347936.f1.pdf]

Supplementary Table 1. Correlation of DPP-IV, sCD26, IgA, IgG and IgM in women from the complete cohort.

	DPP4	sCD26	IgA	IgG	IgM
DPP4 R	1	**0.246****	0.222	-0.103	0.031
p-value		**0.003**	0.129	0.482	0.833
N	146	**144**	48	49	49
sCD26 R		1	-0.022	-0.116	0.065
p-value			0.884	0.428	0.657
N		144	48	49	49
IgA R			1	**0.350***	0.238
p-value				**0.015**	0.103
N			48	**48**	48
IgG R				1	**0.692****
p-value					**<0.0001**
N				49	**49**
IgM R					1
p-value					
N					49

R: Pearson correlation coefficient

Supplementary Table 2. Correlation of DPP-IV, sCD26, IgA, IgG and IgM in men from the complete cohort.

	DPP4	sCD26	IgA	IgG	IgM
DPP4 R	1	0.048	-0.067	0.023	0.028
p-value		0.524	0.567	0.846	0.812
N	182	181	75	75	75
sCD26 R		1	**-0.232***	-0.164	-0.058
p-value			**0.044**	0.158	0.621
N		189	**76**	76	76
IgA R			1	**0.462****	**0.279***
p-value				**<0.0001**	**0.015**
N			76	**76**	**76**
IgG R				1	**0.599****
p-value					**<0.0001**
N				76	76
IgM R					1
p-value					
N					76

R: Pearson correlation coefficient

Supplementary Table 3. Correlation of DPP-IV, sCD26, IgA, IgG and IgM in age group ≤50 years from the complete cohort.

	DPP4	sCD26	IgA	IgG	IgM
DPP4 R	1	**0.200***	0.027	-0.039	-0.078
p-value		**0.026**	0.850	0.782	0.577
N	126	**124**	53	54	54
sCD26 R		1	-0.078	-0.212	0.051
p-value			0.574	0.120	0.712
N		126	54	55	55
IgA R			1	**0.422****	**0.355****
p-value				**0.001**	**0.008**
N			54	**54**	**54**
IgG R				1	**0.660****
p-value					**<0.0001**
N				55	**55**
IgM R					1
p-value					
N					55

R: Pearson correlation coefficient

Supplementary Table 4. Correlation of DPP-IV, sCD26, IgA, IgG and IgM in age group 51-60 years from the complete cohort.

		DPP4	sCD26	IgA	IgG	IgM
DPP4	R	1	**0.254***	-0.194	**-0.347***	-0.125
	p-value		**0.014**	0.230	**0.028**	0.441
	N	95	**94**	40	**40**	40
sCD26	R		1	-0.114	-0.156	0.048
	p-value			0.484	0.335	0.770
	N		94	40	40	40
IgA	R			1	**0.385***	0.311
	p-value				**0.014**	0.051
	N			40	**40**	40
IgG	R				1	**0.746****
	p-value					**<0.0001**
	N				40	**40**
IgM	R					1
	p-value					
	N					40

R: Pearson correlation coefficient

Supplementary Table 5. Correlation of DPP-IV, sCD26, IgA, IgG and IgM in age group ≥61 years from the complete cohort.

	DPP4	sCD26	IgA	IgG	IgM
DPP4 R<br>p-value<br>N	1<br><br>50	0.100<br>0.490<br>50	0.148<br>0.442<br>29	**0.386***<br>**0.038**<br>**29**	0.205<br>0.285<br>29
	sCD26 R<br>p-value<br>N	1<br><br>51	-0.294<br>0.121<br>29	0.098<br>0.612<br>29	-0.185<br>0.337<br>29
		IgA R<br>p-value<br>N	1<br><br>29	**0.574****<br>**0.001**<br>**29**	0.219<br>0.254<br>29
			IgG R<br>p-value<br>N	1<br><br>29	0.340<br>0.071<br>29
				IgM R<br>p-value<br>N	1<br><br>29

R: Pearson correlation coefficient
